# Mechanism of Activation and Microstructural Evolution in Calcium Carbide Slag-Activated GGBS-CG Composite Cementitious Materials

**DOI:** 10.3390/ma18174189

**Published:** 2025-09-06

**Authors:** Tengfei Wang, Feng Ju, Meng Xiao, Dong Wang, Lidong Yin, Lu Si, Yingbo Wang, Mengxin Xu, Dongming Yang

**Affiliations:** State Key Laboratory of Intelligent Construction and Healthy Operation and Maintenance of Deep Underground Engineering, China University of Mining and Technology, Xuzhou 221116, China; wangtf0623@cumt.edu.cn (T.W.); m.xiao@cumt.edu.cn (M.X.); mechanics@cumt.edu.cn (D.W.); yinlidong532@163.com (L.Y.); ts23030012a31@cumt.edu.cn (L.S.); 15255791521@163.com (Y.W.); ts23030019a31@cumt.edu.cn (M.X.); dongmingyang11@outlook.com (D.Y.)

**Keywords:** calcium carbide slag, ground granulated blast-furnace slag, coal gangue, alkaline activation, mechanical property, microstructural evolution

## Abstract

The efficient resource utilization of industrial solid wastes, such as ground granulated blast-furnace slag (GGBS) and coal gangue (CG), is essential for sustainable development. However, their activation commonly depends on expensive and corrosive chemical alkalis. This study proposes a solution by developing a fully waste-based cementitious material using calcium carbide slag (CS), another industrial residue, as an eco-friendly alkaline activator for the GGBS-CG system. The influence of CS dosage (0–20 wt%) on hydration evolution and mechanical properties was examined using uniaxial compression testing, X-ray diffraction (XRD), Fourier-transform infrared spectroscopy (FTIR), and scanning electron microscopy (SEM). The results indicated that a CS dosage of 10 wt% yielded the highest compressive strength, reaching 10.13 MPa—a 16.5% improvement compared to the 20 wt% group. This enhancement is ascribed to the formation of hydrotalcite (HT) and calcium silicate hydrate (C-(A)-S-H) gel, which densify the microstructure. In contrast, higher CS contents led to a passivation effect that restrained further reaction. This work offers a practical and theoretical basis for the development of low-carbon, multi-waste cementitious materials and presents a promising strategy for large-scale valorization of industrial solid wastes.

## 1. Introduction

The efficient utilization of industrial solid wastes is a key challenge in advancing sustainable materials’ development [1,2]. In China, coal gangue (CG) and ground granulated blast-furnace slag (GGBS), major by-products of coal mining and metallurgical industries, generate annual emissions exceeding 700 million tons and 300 million tons, respectively. Long-term stockpiling of these wastes consumes valuable land resources and poses environmental risks, including heavy metal leaching, particulate emissions, and spontaneous combustion hazards [3,4,5]. Although traditional cementitious materials, such as ordinary Portland cement, are conventionally used in mine backfilling, their production is energy-intensive and contributes approximately 8% of global CO_2_ emissions. Consequently, developing low-carbon cementitious systems primarily composed of solid wastes has become a critical research frontier, offering a promising alternative for sustainable mine backfilling and other construction applications.

Due to their high alumina and silica content and activation potential, industrial wastes like GGBS and CG have been extensively studied as precursors for geopolymer synthesis [6,7,8,9]. Öz A et al.’s study utilized a Na_2_SiO_3_/NaOH composite activator (NS/NH ratios: 1.5–3.0) to activate ground granulated blast furnace slag (GGBS) blended with waste materials (brick powder, zeolite, or rice husk ash). Optimizing the NS/NH ratio to 2.5 under 80 °C curing maximized geopolymerization of slag, forming dense C-A-S-H gel networks that achieved peak compressive strength (44.58 MPa) and minimized porosity/sorptivity [10]. Kurtay-Yildiz et al., using a Na_2_SiO_3_/NaOH activator (NS/NH = 2.5, 10 M NaOH), activated GGBS-ceramic waste powder (CWP) blends hybrid-reinforced with PVA/steel fibers. Critical finding: 0.7% PVA + 1.3% steel fibers synergistically enhanced long-term performance, achieving 118.7 MPa compressive strength at 365 days (+11% vs. non-fibrous) and 85× higher fracture energy, demonstrating superior crack resistance [11]. Huahui Yin et al.’s study utilized NaOH-Na_2_SiO_3_ alkali activators to stimulate a ternary precursor system comprising spontaneous combustion coal gangue (SCG), ground granulated blast furnace slag (GGBFS), and waste ceramic powder (WCP), achieving peak 28-day compressive strength (94.1 MPa) at 5% Na_2_O activator content due to optimized C-(A)-S-H gel formation [12]. Qiao et al. found that NaOH (optimally at 5 mol/L) effectively activated an SS-FA-GGBFS composite to form strength-enhancing C-S-H/C-A-H gels, whereas Na_2_CO_3_ activation resulted in weaker calcium carbonate formation and long-term strength deterioration [13].

Yang et al.’s study utilized sodium silicate with varying moduli and alkali contents to optimize the activator system, successfully producing high-performance coal gangue-based geopolymers by activating pre-calcined CG (700 °C for 2 h) and GGBS [14]. Zhang et al. [15] utilized NaOH, KOH, and sodium silicate activators to prepare CG-based geopolymers, identifying amorphous alkali aluminosilicate gels resembling zeolite precursors. Han et al. [16] demonstrated that sodium silicate-activated CG-GGBS composites achieve compressive strengths exceeding 40 MPa with CG content below 30 wt%. Ma et al. [17] identified the liquid-to-solid ratio as the primary factor influencing workability and strength in NaOH/sodium silicate-activated CG-GGBS systems (AACGS). Geng et al. [18] explored the synthesis of coal gangue-based geopolymers employing a composite alkaline activator system of sodium hydroxide (5 mol/L) and water glass (modulus 3.4, 66 wt% water content). Through a combination of mechanical property evaluation and advanced microscopic characterization, they systematically uncovered the influence of activator ratios on the microstructure, mineral composition, and crystalline structure of the geopolymers.

However, the high cost and corrosivity of conventional alkaline activators (e.g., NaOH, Na_2_SiO_3_) limit their industrial applicability. Calcium carbide slag (CS), a Ca(OH)_2_-rich by-product from acetylene production with an annual output exceeding 30 million tons [19], offers a sustainable alternative due to its strong alkalinity (pH > 12) and high calcium content. Yang et al. [20] reported improved mechanical properties and hydration products in fly ash geopolymers using CS/sodium silicate hybrid activators. Shi et al. [21] developed all-solid-waste geopolymers using CS as an activator, confirming its efficacy in promoting raw material dissolution. Hanjitsuwan et al. [22] observed accelerated setting and strength development in CS-modified alkali-activated fly ash mortars. Li et al. [23] confirmed that Ca(OH)_2_ in CS enhances GGBS reactivity, generating abundant hydration products. These studies collectively validate CS as an effective alkaline activator. Existing research primarily focuses on CS activation in single-component systems (e.g., GGBS or fly ash) [24].

Despite these advancements, a significant technological challenge remains. The low reactivity of raw coal gangue (CG) often necessitates the use of CS in conjunction with expensive chemical activators (e.g., NaOH, Na_2_SiO_3_) to achieve effective activation in hybrid systems [25]. This requirement undermines the economic and environmental benefits of utilizing solid wastes. Furthermore, while the individual activation of GGBS or the hybrid activation of pre-calcined CG has been explored, the synergistic mechanisms within a fully waste-based ternary system (GGBS-CG-CS) are still poorly understood. Specifically, the influence of CS dosage on the reaction pathways, microstructure evolution, and role of inert phases from CG remain underexplored. This gap in knowledge hinders the optimization and predictable application of such multi-waste systems.

This study establishes a GGBS-CG composite system as the matrix. By systematically varying the CS dosage (0–20 wt%), combined with 7-day uniaxial compression testing, XRD, FTIR, and SEM characterization, we investigate the dual influence of CS on early-age mechanical properties and microstructural evolution. The findings provide a theoretical basis for optimizing the performance and industrial application of solid waste-based cementitious materials.

## 2. Materials and Methods

### 2.1. Raw Materials

**Figure 1:**
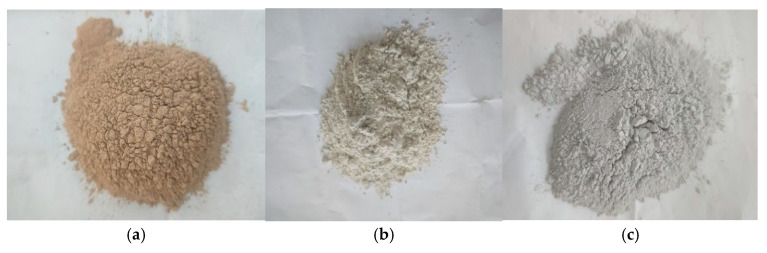
Field images of raw materials: (**a**) CG, (**b**) GGBS, (**c**) CS.

**Figure 2:**
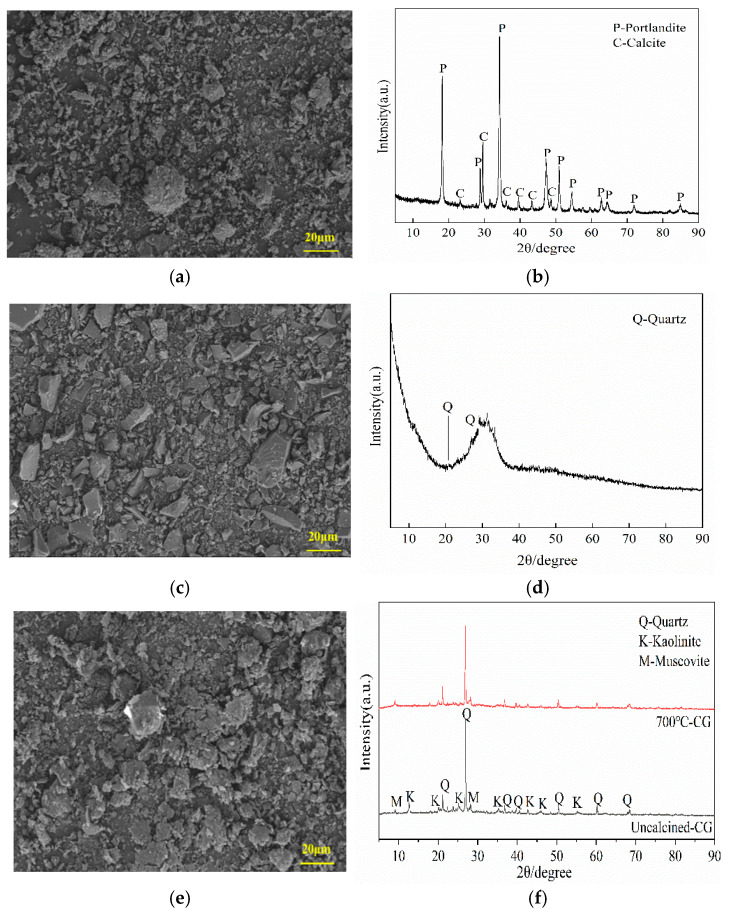
SEM image of (**a**) CS, (**c**) GGBS, and (**e**) CG; and XRD spectra of (**b**) CS, (**d**) GGBS, and (**f**) CG.

**Figure 3:**
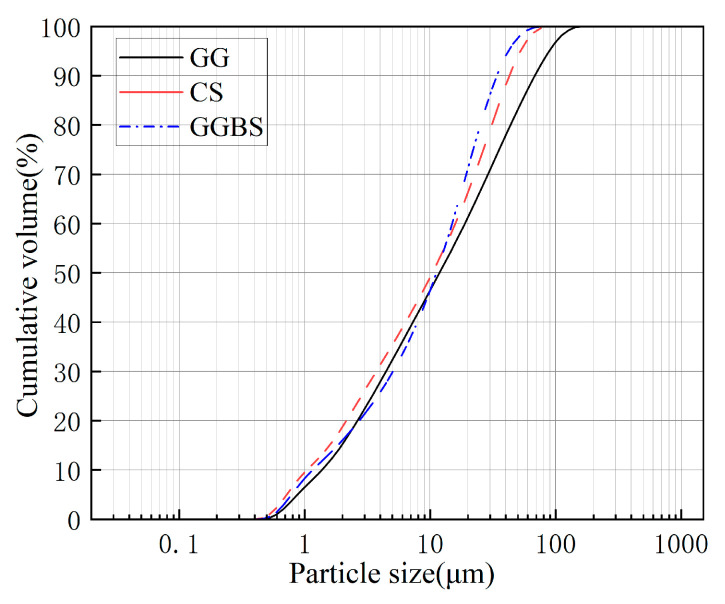
Particle size distributions of CG, GGBS, and CS.

CG: Sourced from Xuzhuang Coal Mine, Jiangsu. The raw material was coarsely crushed using a jaw crusher, followed by ball milling in a planetary ball mill for 2 h (ball-to-material mass ratio of 5:1, rotational speed of 300 rpm). It was then calcined in a muffle furnace at 700 °C for 2 h and sieved through a 0.15 mm square-hole sieve. The sample exhibited a pale yellow color (Figure 1a) with an irregular blocky microstructure (Figure 2e). X-ray fluorescence (XRF) analysis indicated primary chemical compositions of SiO_2_ (46.55 wt%) and Al_2_O_3_ (44.46 wt%), with minor amounts of Fe_2_O_3_ (2.71 wt%), K_2_O (0.68 wt%), and others (Table 1). X-ray diffraction (XRD) phase analysis (Figure 2f) confirmed the presence of quartz, kaolinite, and muscovite. (1) After thermal activation (700 °C for 2 h), the characteristic diffraction peak intensity of kaolinite significantly decreased, indicating effective disruption of its lattice structure; (2) Quartz and muscovite diffraction peaks showed no significant shifts or intensity changes, confirming their structural stability, while calcination markedly enhanced the reactivity of CG [26]. Ground Granulated Blast Furnace Slag (GGBS): Conforming to S95 grade per GB/T 18046-2017 [27]. The sample appeared grayish-white (Figure 1b) with an irregular blocky microstructure (Figure 2c). XRF analysis revealed primary components of CaO (39.50 wt%), SiO_2_ (32.80 wt%), and Al_2_O_3_ (14.30 wt%) (Table 1), with trace amounts of quartz crystals (Figure 2d). Calcium Carbide Slag (CS): Obtained as a byproduct from a PVC plant in Inner Mongolia. The material was dried at 105 °C for 24 h and sieved through a 200-mesh screen. The sample exhibited a bluish-gray color (Figure 1c) with an orthorhombic, irregular blocky microstructure (Figure 2a). XRF analysis indicated a primary composition of CaO (67.20 wt%), with minor amounts of MgO (0.22 wt%), SiO_2_ (2.56 wt%), and others (Table 1). XRD analysis confirmed the phase composition as portlandite (Ca(OH)_2_) and calcite (CaCO_3_) (Figure 2b). Figure 3 shows the particle size distributions of CG, GGBS, and CS, with median particle sizes of 18.4 µm, 15.7 µm, and 7.49 µm, respectively.

### 2.2. Mix Proportion Design

The GGBS and CG composite system served as the cementitious base, with a fixed mass ratio of GGBS:CG = 40:60. CS was incorporated by partially replacing the base material using the internal blending method, with designed CS dosage gradients of 0 wt%, 5 wt%, 10 wt%, 15 wt%, and 20 wt% (by total mass of the cementitious material). The specific mix proportions are presented in Table 2.

### 2.3. Sample Preparation and Curing

First, mix CG-GGBS-CS in different proportions, and stir them in a cement mixer for 5 min. Then, add varying amounts of ultrapure water to the cement mixer to blend with the solid raw materials, forming a uniform paste. Inject each mixed paste into cylindrical molds (ϕ50 × 100 mm), use a geometry specified by the International Society for Rock Mechanics (ISRM) for uniaxial compression tests on rock-like materials [28], vibrate for 2 min to remove air bubbles, and then cover all filled silicone molds with a thin polyethylene film to prevent moisture evaporation. The filled molds were then placed in the laboratory environment at a temperature of approximately 31 °C for 24 h before demolding. After demolding, the samples were transferred to a standard constant temperature and humidity curing chamber (20 ± 1 °C, RH ≥ 95%) for 7 days of further curing [29]. Four replicate specimens were prepared for each mix proportion. After curing, three specimens were used for uniaxial compression tests, and the fourth was reserved for microstructural characterization (XRD, FTIR, and SEM).

### 2.4. Testing Methods

Uniaxial Compressive Strength (UCS): Tests were conducted using an MTS816 hydraulic servo testing machine(MTS Systems Corporationin in Eden Prairie, Minnesota, United States), in accordance with the general principles of standard ASTM C39/C39M [30], in displacement control mode at a constant loading rate of 0.3 mm/min. For each mix proportion, three cylindrical specimens were tested after 7 days of curing. Data with a deviation greater than 5% were excluded, and the arithmetic mean was calculated from the remaining values.

Sample Preparation for Microstructural Analysis: The fourth specimen from each group was broken after 7 days of curing. Fragments from the interior of the specimen were collected to avoid any skin effects. The hydration reaction was immediately arrested for these samples to preserve the microstructure at the desired age. For XRD and FTIR analysis, the fragments were crushed and ground. The powder was then immersed in isopropanol for 24 h to stop hydration, followed by drying in a vacuum oven at 60 °C for 24 h. For SEM analysis, small, flat pieces (approximately 5 × 5 × 5 mm) were taken from the fresh fracture surface and immediately immersed in anhydrous ethanol for 24 h, then dried in a vacuum desiccator for 24 h.

X-Ray Diffraction (XRD): The mineral phases were analyzed using a Bruker D8 Advance diffractometer. The sample preparation and testing procedure followed the guidelines of ASTM C1365 for the analysis of cementitious materials. The prepared dry powder was passed through a 0.075 mm sieve to ensure fineness. The measurement was performed with a scanning range of 5–70° (2θ), a step size of 0.02°, and a scanning rate of 2°/min. Phase identification was performed using Jade 6.5 software.

Fourier-Transform Infrared Spectroscopy (FTIR): FTIR spectroscopy was conducted using a Bruker Vertex 80v vacuum spectrometer by employing the standard KBr pellet method. The dried powder was passed through a 0.15 mm sieve. The spectra were collected covering a spectral range of 400–4000 cm^−1^ with a resolution of 0.06 cm^−1^ and 64 cumulative scans. Spectral data were analyzed using Omnic 9.2 software.

Scanning Electron Microscopy (SEM): Morphological analysis was performed using a ZEISS-Sigma 360 field-emission SEM (Zeiss, Oberkochen, Germany). Prior to testing, samples were prepared following the standard protocol for non-conductive materials; the dried samples were mounted on aluminum stubs and sputter-coated with a thin layer of gold to enhance conductivity prior to imaging.

## 3. Results and Discussion

### 3.1. Compressive Strength Test

**Figure 4:**
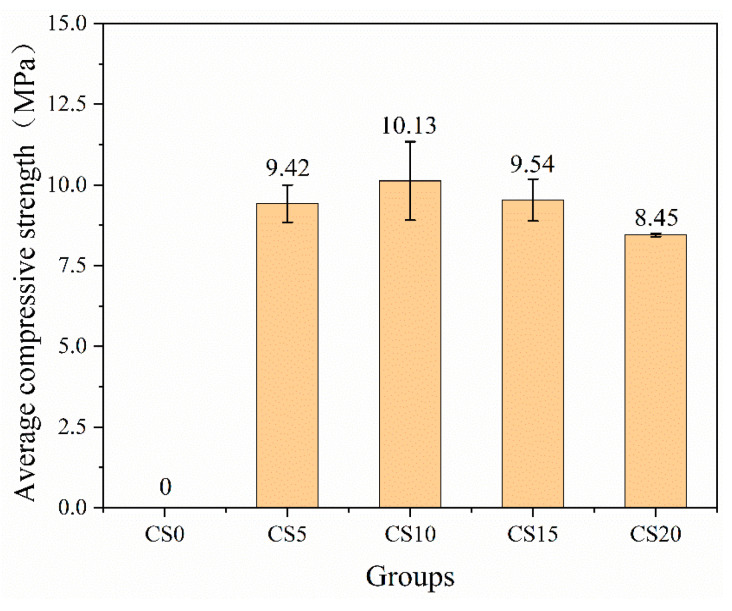
Compressive strength of samples.

The results of the unconfined uniaxial compressive strength tests are shown in Figure 4. The control group with 0 wt% CS exhibited a loose structure due to the absence of effective hydration products for binding, making it impossible to prepare standard specimens for mechanical testing. Macroscopically, this manifested as a complete lack of unconfined compressive strength. This phenomenon confirms that, without alkaline activation, GGBS and CG do not undergo significant hydration reactions. When the CS content was increased to 5 wt%, the specimen achieved a compressive strength of 9.42 MPa, indicating that CS, acting as an alkaline activator, effectively triggered the hydration reactions of GGBS and CG. The compressive strength exhibited a trend of initially increasing and then decreasing with higher CS content. At a CS content of 10 wt%, the compressive strength peaked at 10.13 MPa, representing a 7.4% increase compared to the 5 wt% CS group. However, as the CS content further increased to 15 wt% and 20 wt%, the strength decreased by approximately 5.8% and 16.5%, respectively. These results suggest that the highest compressive strength was achieved at a CS dosage of 10 wt%. Three potential reasons may explain the decline in strength after the peak: (1) Higher CS content increases the formation of portlandite crystals, which may suppress strength development [31,32]. (2) As CS content rises, it displaces more GGBS and CG, which are the primary raw materials for the polymerization reaction, leading to insufficient reactants [25]. (3) Excessive CS results in an overly high OH^−^ concentration, promoting the premature formation of a protective layer of hydration products on the surfaces of some GGBS and CG particles, which hinders further hydration and limits strength enhancement [33,34].

### 3.2. XRD Analysis

**Figure 5:**
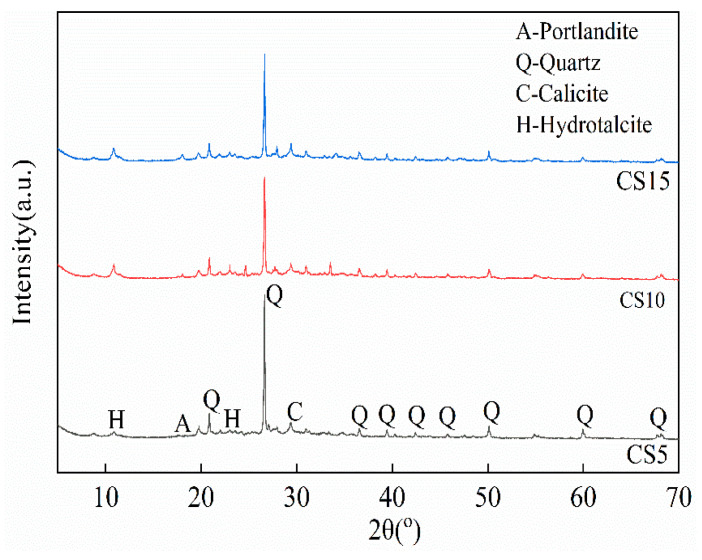
XRD patterns of samples.

To investigate the hydration products of the cementitious materials and elucidate the impact of CS content on these products, XRD patterns of the CS5, CS10, and CS15 samples were obtained and analyzed after 7 days of curing. The XRD patterns reveal that the primary phases in all three samples are Quartz, Portlandite, Calcite, and Hydrotalcite. The formation of Hydrotalcite results from the re-polymerization reaction of Mg^2+^ ions dissolved from GGBS with amorphous aluminum hydroxide under high pH conditions [35]. Due to its unique layered structure, Hydrotalcite crystals effectively fill voids, enhancing matrix density and improving mechanical properties. Quartz originates from GGBS and CG, Portlandite is derived from CS, and Calcite likely forms due to the carbonation of CS [36]. As the CS content increases from 5 wt% to 10 wt%, the diffraction peak intensity of Hydrotalcite significantly strengthens, indicating that an appropriate CS content accelerates the hydration rate of GGBS and CG, increasing the formation of crystalline Hydrotalcite. However, when the CS content rises to 15 wt%, the Hydrotalcite peak intensity decreases, and the hydration rate slows, likely due to excessive OH^−^ suppressing the hydration reaction and reducing Hydrotalcite formation. In this experimental design, the Quartz peak intensity in the CS10 sample is lower than that in the CS15 sample, suggesting that more Quartz in the GGBS and CG participates in the hydration reaction in the CS10 sample, indicating a higher degree of reaction compared to the CS15 group. The C-(A)-S-H gel, an amorphous hydration product, appears as a diffuse peak in the XRD patterns [25,37,38]. In Figure 5, a “hump” is observed in the 20–40° range across all patterns, confirming the presence of amorphous C-(A)-S-H gel. The CS10 sample exhibits the highest gel content, which contributes to enhanced uniaxial compressive strength of the cementitious material [39]. In the CS15 sample, the Portlandite peak intensity is the highest, attributed to the large incorporation of CS, which suppresses the hydration reaction, leaving a significant amount of unreacted Portlandite. Additionally, due to unavoidable exposure to atmospheric CO_2_ during mixing and curing, Portlandite in the cementitious material reacts with CO_2_ to form Calcite.

### 3.3. FTIR Analysis

**Figure 6:**
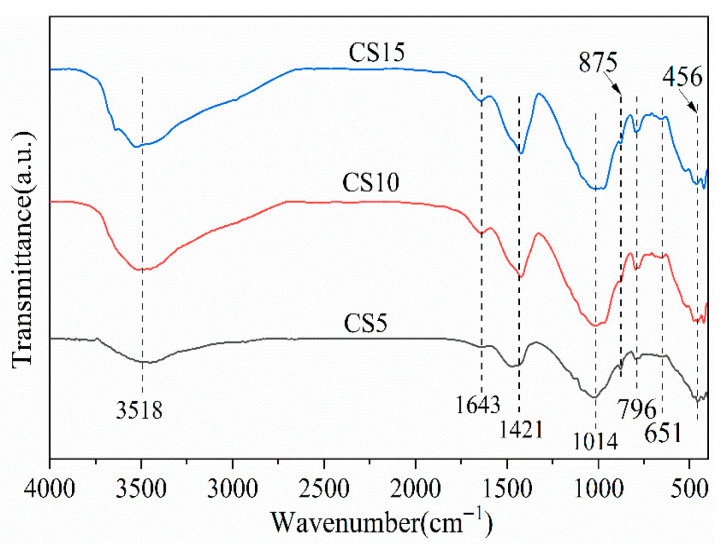
FTIR spectra of samples.

To elucidate the influence of CS content on the chemical structure of hydration products, FTIR spectroscopy analysis was performed on the CS5, CS10, and CS15 samples (Figure 6). The evolution of key spectral bands is summarized as follows: Si-O-Si Vibration Peak Shifts: The absorption bands near 456 cm^−1^, 796 cm^−1^, and 1014 cm^−1^ correspond to the in-plane bending vibration, out-of-plane bending vibration, and stretching vibration of Si-O bonds, respectively, which are characteristic peaks of C-(A)-S-H gel [40,41]. The shift and broadening of the band near 1014 cm^−1^ are particularly noteworthy, indicating the incorporation of Al^3+^ into the silicate chains to form a C-A-S-H type gel. This is primarily facilitated by the high Al_2_O_3_ content (~44.46 wt%) in coal gangue (CG), which provides a substantial source of aluminum for the reaction. The peak intensity and width of these bands follow the order: CS10 > CS15 > CS5. This indicates that with the incorporation of CS into the GGBS-CG cementitious system, the transmittance of Si-O bond absorption bands decreases, and the bands shift to higher wavenumbers. This suggests an increase in the polymerization degree of silicate anions in the hydration products [42], with the highest C-(A)-S-H gel formation observed in the CS10 sample. However, excessive CS content hinders silica polycondensation, reducing C-(A)-S-H gel formation. Carbonate-Related Peaks: The absorption peaks near 875 cm^−1^ and 1421 cm^−1^ correspond to the out-of-plane bending vibration of CO_3_^2−^ [40] and the symmetric stretching vibration of C-O bonds [43], respectively, attributed to Calcite and Hydrotalcite (HT) [44]. Water-Related Peaks: The absorption peaks near 1643 cm^−1^ and 3518 cm^−1^ are associated with the bending vibration and asymmetric stretching vibration of H-O bonds, respectively, representing the internal vibrational absorption of crystal water [45]. This crystal water primarily originates from the hydroxyl groups in the C-(A)-S-H gel. The peak intensity and width of these bands are highest in the CS10 sample, indicating the lowest transmittance and the greatest formation of hydration products containing crystal water, followed by CS15 sample and CS5 sample. These findings further corroborate the XRD results.

### 3.4. SEM Analysis

**Figure 7:**
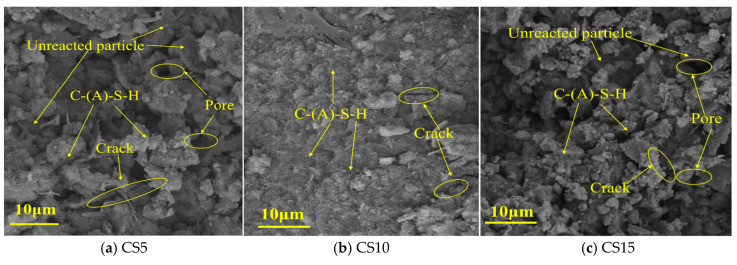
The SEM images analysis results of CS5, CS10, CS15 samples.

The SEM images in Figure 7 reveal significant microstructural degradation in samples with 5 wt% and 15 wt% CS content. For the CS5 sample (Figure 7a), the low CS content results in insufficient OH^−^ concentration, which hinders hydration kinetics and limits the formation of C-(A)-S-H gel. Consequently, numerous unreacted particles and interconnected microcracks propagate through micropores, forming macroscopic defects. In contrast, the CS15 sample (Figure 7c) exhibits excessive initial alkalinity, causing coal gangue and slag particles to be partially encapsulated by rapidly precipitated C-(A)-S-H, which inhibits further hydration. This leads to a well-developed network of pores and cracks, compromising structural integrity. By contrast, the CS10 sample (Figure 7b) displays a dense and uniform microstructure characterized by abundant C-(A)-S-H gel binding unreacted particles. Only isolated microcracks are observed, with no interconnected defects. These microstructural observations align with the uniaxial compressive strength results, indicating that 10 wt% CS provides the most effective alkaline activation: it balances OH^−^ concentration to maximize hydration efficiency while avoiding the development of porosity and reaction barriers.

### 3.5. Mechanism of Strength Formation in the Cementitious System

Based on the comprehensive microscopic analysis and with reference to previous research on the strength formation mechanisms of alkali-activated cementitious materials [36], the hydration process of calcium carbide slag-activated slag-coal gangue cementitious material can be described as follows. Combined with Equations (1)–(5), the strength formation mechanism of this novel cementitious system can be described in the following three stages:

1. Raw Material Dissolution: Calcium carbide slag establishes a highly alkaline environment. Initially, the Ca–O and Mg–O bonds, which have relatively weaker bond energies in the slag glassy structure, break first, releasing Ca^2+^ and Mg^2+^ ions. Subsequently, the stronger Al–O and Si–O bonds in the glassy structures of both the slag and coal gangue begin to break down under the attack of OH^−^ ions, forming silicate and aluminate ionic species ([H_3_SiO_4_]^−^, [H_3_AlO_4_]^2−^, [Al(OH)_6_]^3−^). The octahedral [Al(OH)_6_]^3−^ is primarily formed from the dissolution of slag. Concurrently, part of the calcium carbide slag dissolves, releasing OH^−^, Ca^2+^, and other ions.
2. Formation of Hydration Products: The [H_3_SiO_4_]^−^ ions in the solution combine with Ca^2+^ to form initial C-S-H gel, while also interacting with [H_3_AlO_4_]^2−^ to form C-A-S-H gel. A small amount of [Al(OH)_6_]^3−^ combines with CO_3_^2−^ and Mg^2+^ to form hydrotalcite (HT, 6MgO·Al_2_O_3_·CO_2_·12H_2_O). As the reaction progresses, the formation of C-(A)-S-H gel and HT continuously consumes [H_3_SiO_4_]^−^, [H_3_AlO_4_]^2−^, [Al(OH)_6_]^3−^, OH^−^, and Ca^2+^, which in turn promotes further dissolution of the raw materials. Furthermore, as water is consumed, a higher CO_2_ content in the environment can lead to the carbonation of Ca(OH)_2_ in the system, forming calcite crystals.
3. Polymerization of Hydration Products: As the dissolution of raw materials and the formation of hydration products proceed continuously, the C-(A)-S-H gel grows steadily, adhering to the surfaces of unreacted particles and binding the dispersed particles into an integrated whole. The primary crystalline product, HT, along with other crystals such as calcite and a small amount of unreacted quartz, are distributed on the gel surface and within existing microcracks, serving to bridge and fill these microcracks. The gel and crystalline hydration products together form the hardened paste skeleton, thereby developing strength.
  SiO_2_ + OH^−^ + H_2_O = [H_3_SiO_4_]^−^ (1)
  AlO_2_^−^ + OH^−^ + H_2_O = [H_3_AlO_4_]^2−^ (2)
  AlO_2_^−^+OH^−^+H_2_O = [Al(OH)_6_]^3−^ (3)
  [H_3_SiO_4_]^−^ + ([H_3_AlO_4_]^2−^) + Ca^2+^ = C-(A)-S-H (4)
  4OH^−^ + CO_3_^2−^ + 6Mg^2+^ + 2[Al(OH)_6_]^3+^ 4H_2_O = 6MgO·Al_2_O_3_·CO_3_·12H_2_O (5)

### 3.6. Discussion

This study demonstrates that calcium carbide slag (CS) serves as an effective alkaline activator for GGBS-CG composites, with its dosage critically controlling both mechanical properties and microstructural development. The compressive strength exhibited a non-monotonic relationship with CS content, peaking at 10.13 MPa with 10 wt% CS before declining at higher dosages. This trend aligns with findings from Li W. et al. [46] and Cong P. et al. [47], confirming the existence of a well-defined activation window in waste-based geopolymer systems. Microstructural analysis revealed that 10 wt% CS provided balanced chemical conditions for aluminosilicate dissolution and reaction product formation. XRD and FTIR confirmed the synergistic formation of C-(A)-S-H gel and hydrotalcite at this dosage, which enhanced matrix density through pore filling and interfacial bonding. SEM observations correlated these findings with a dense, homogeneous microstructure in the 10 wt% CS sample. Compared to traditional activators like NaOH or Na_2_SiO_3_, CS offers sustainable advantages through its dual function as a source of both alkalinity and calcium ions [48]. The gradual release of OH^−^ from portlandite dissolution may enable more controlled reaction kinetics compared to the rapid pH spike from soluble alkalis. These findings provide valuable insights into the activation mechanisms of waste-based cementitious materials and establish CS as a viable sustainable activator for multi-component systems. The identified CS dosage of 10 wt% represents an efficient balance between activation efficiency and material consumption, highlighting the potential for practical applications in sustainable construction.

## 4. Conclusions

This study utilized a GGBS–CG composite system as the matrix, with gradient adjustments of CS content (5–20 wt%). Through uniaxial compression tests, XRD, FTIR and SEM characterization, the following conclusions were drawn:

1. CS content critically regulates the alkaline activation process. A dosage of 10 wt% was identified as the most effective among the tested formulations, providing a chemical environment that maximizes aluminosilicate dissolution and promotes the synergistic formation of C-(A)-S-H gel and hydrotalcite, leading to effective pore filling and matrix densification.
2. Both mechanical performance and microstructural evolution are governed by the balance between activation and precursor availability. Excessive CS content disrupts this balance through passivation effects and reduction of reactive components, resulting in strength decline and microstructural degradation.

These findings establish CS as an effective microstructural modulator in multi-waste cementitious systems and provide a scientific basis for optimizing solid waste-based activators in sustainable construction materials. From a practical perspective, this work offers a viable pathway for large-scale utilization of industrial by-products in medium-strength applications such as road bases, backfills, and prefabricated elements.

Future work will employ advanced statistical methods for mixture optimization, elucidate reaction kinetics via isothermal calorimetry, apply advanced quantitative characterization techniques (e.g., quantitative phase analysis by XRD and FTIR, statistically representative microstructural analysis), and evaluate long-term performance including durability (e.g., sulfate resistance, freeze–thaw cycling) and strength evolution beyond 7 days.

## Figures and Tables

**Table 1:** Chemical composition of raw materials (wt.%).

Material	Chemical Composition by Weight (wt.%)								
	SiO_2_	Al_2_O_3_	CaO	MgO	Fe_2_O_3_	Na_2_O	K_2_O	MnO	SO_3_
CS	2.56	1.68	67.20	0.22	0.09	0.35	0.03	/	0.68
GGBS	32.80	14.30	39.50	9.20	0.88	0.20	0.63	0.07	1.32
CG	46.55	44.46	2.98	0.52	2.71	0.12	0.68	0.12	0.31

**Table 2:** Mix proportions of CG-GGBS-CS composites.

Groups	GG (wt%)	GGBS (wt%)	CS% (wt%)	Water-to-Binder Ratio (W/B)	Si/Al
CS0	60%	40%	0%	0.5	1.08
CS5	57%	38%	5%	0.5	1.08
CS10	54%	36%	10%	0.5	1.08
CS15	51%	34%	15%	0.5	1.08
CS20	48%	32%	20%	0.5	1.08

## Data Availability

The original contributions presented in this study are included in the article. Further inquiries can be directed to the corresponding author.
